# Advancements in Host-Based Interventions for Influenza Treatment

**DOI:** 10.3389/fimmu.2018.01547

**Published:** 2018-07-10

**Authors:** Tsz-Fung Yip, Aisha Sami Mohammed Selim, Ida Lian, Suki Man-Yan Lee

**Affiliations:** ^1^HKU-Pasteur Research Pole, School of Public Health, The University of Hong Kong, Hong Kong, Hong Kong; ^2^School of Life Sciences and Chemical Technology, Ngee Ann Polytechnic, Singapore, Singapore

**Keywords:** host factors, influenza, cytokines, metabolism, immunomodulation

## Abstract

Influenza is a major acute respiratory infection that causes mortality and morbidity worldwide. Two classes of conventional antivirals, M2 ion channel blockers and neuraminidase inhibitors, are mainstays in managing influenza disease to lessen symptoms while minimizing hospitalization and death in patients with severe influenza. However, the development of viral resistance to both drug classes has become a major public health concern. Vaccines are prophylaxis mainstays but are limited in efficacy due to the difficulty in matching predicted dominant viral strains to circulating strains. As such, other potential interventions are being explored. Since viruses rely on host cellular functions to replicate, recent therapeutic developments focus on targeting host factors involved in virus replication. Besides controlling virus replication, potential targets for drug development include controlling virus-induced host immune responses such as the recently suggested involvement of innate lymphoid cells and NADPH oxidases in influenza virus pathogenesis and immune cell metabolism. In this review, we will discuss the advancements in novel host-based interventions for treating influenza disease.

## Introduction

Influenza remains a source of public health concern. Influenza A virus (IAV) has been the cause of historical noxious pandemics, such as the Spanish flu 1918 H1N1, Asian flu H2N2 1957, Hong Kong H3N2 flu 1968, and more recently the pandemic of H1N1 2009 (Swine flu). Influenza also causes seasonal epidemics and outbreaks with high morbidity and mortality rates such as the 2015 H1N1 outbreak in India (1, 2). The error-prone nature of the viral RNA polymerase (RdRP) and virus’ capacity for genetic re-assortment (antigenic drift and shift) result in the viral components’ susceptibility to mutations, allowing the viruses to evade the immune system and increases their resistance to control strategies.

Currently, influenza vaccination and two classes of antiviral drugs—M2 ion channel blockers (amantadine and rimantadine) and neuraminidase (NA) inhibitor (oseltamivir, zanamivir, and peramivir)—and the novel treatment option using polymerase inhibitor (favipiravir) are considered as mainstays in influenza infection treatment and control. The use of influenza vaccinations remains challenging due to antigenic drifts and shifts, with seasonal variation of new circulating species. Production of vaccine is time consuming with efficacy concerns, especially in the case of pandemic. Variations in vaccine efficacy caused by age should be aware, with studies suggesting that vaccine-conferred protection may not be optimal in certain age groups (3).

The disadvantages of using the conventional antiviral drugs have also been a concern. Significant levels of resistance to both classes of drugs have been repeatedly reported (4, 5). High level of resistance (up to 91%) to M2 blockers has been reported in H3N2 virus strain in American isolates (6). Resistance has also been reported in H5N1 virus (7). IAV resistance to NA inhibitors has also become an increasingly prevalent concern, with the recent highly fatal outbreak of influenza A(H1N1)pdm09 in India 2015 associated with oseltamivir drug resistance (8, 9). In addition, a large cluster of influenza A(H1N1)pdm09 viruses in Japan was found to have increased oseltamivir and peramivir drug resistance (5). There is an urgent need to search for alternative targets to treat influenza virus infections, including non-viral targets such as host cellular factors; which are promising as viruses rely on the host machinery for replication. While host immune response is intended to confer a degree of protection against the infection, an impaired or exaggerated host immune response could be detrimental—IAV H5N1 and H7N9 virus infection was reported to exaggerate aberrant cytokine release, resulting in a cytokine storm that caused accelerated host death (10–12).

Many recent studies have focused on the investigation of targeting host factors to control virus replication as well as modulate immune response, which we have previously evaluated (13). In this review, we will discuss the latest studies (in the past 5 years) on the investigation of novel host-based approaches with potential for influenza treatment.

## Strategies Targeting Host Cell Machinery

The replication cycle of IAV can be grossly divided into four different stages: (1) entry, (2) genome nuclear import, (3) replication and protein synthesis, and (4) genome nuclear export, apical transport, assembly, and budding. As an obligate intracellular pathogen, IAVs are heavily dependent on host machinery for replication and propagation. To this extent, studies employing genome-wide RNA interference (RNAi) to screen for host factors involved in IAV replication cycle have been performed (14, 15) and an increasing number of approaches targeting these host factors to control IAV replication have been investigated.

### Entry of IAV

Entry of IAV into the host cell is divided into several steps (16, 17). First, hemagglutinin (HA) on the surface of IAV binds to the terminal α-sialic acid on the host cell receptor. This induces the internalization of the viral particle by clathrin-dependent, caveolin-, and clathrin-independent endocytosis (18). Macropinocytosis was revealed as an alternative entry pathway for IAV (19), which subsequently enters the canonical endocytic pathway (20, 21). The vesicle-containing viral particle forms an early endosome (also known as sorting endosome), which matures into a late endosome as the endocytic pathway progresses. A gradual decrease in intraluminal pH from pH 6.5 to 5.0, mediated by V-ATPase proton pump (22), takes place as the endosome matures (23, 24). This pH drop in the endosomal lumen induces a conformational change in HA, which is activated by proteolytic cleavage to generate HA1 and HA2 from precursor molecule HA0 (25, 26). This conformational change triggers the fusion of the viral envelope with the endosomal membrane, releasing the viral genome into the cytoplasm.

Acidification of the endosome causes the subsequent acidification of viral lumen *via* the IAV M2 proton channel (27), which in turn promotes the dissociation of M1 layer from both the viral envelope (24) and the viral ribonucleoprotein (vRNP) complex (28). Interestingly, a sharp decrease in pH from neutral to an acidic pH of 5.0 as utilized by acid bypass has been observed to be sub-optimal for viral replication. It is hence proposed that a gradual decrease in endosomal pH is necessary for sequential reduction in viral stiffness, dissociation of M1 from the NP in the vRNP complex, destabilization of M1 layer from the viral envelope, and the eventual conformational change of the HA for the release of viral genome and proteins to the cytoplasm from late endosome (24).

#### Inhibition of Proteolytic Cleavage of HA

Proteolytic cleavage of HA0 to HA1/HA2 is an important step in IAV replication. This cleavage relocates HA2, converting previously uncleaved HA0 to a metastable conformation that induces membrane fusion at acidic pH (29). Inefficient cleavage and activation of HA leads to low infectivity (30). As identified proteins encoded by the viral genome do not possess proteolytic properties, the virus is dependent on host protease for the cleavage of HA. This provides a potential target to control IAV infection. HA is commonly cleaved by trypsin-like proteases at the single arginine residue at position 329. Human airway epithelium serine proteases HAT and TMPRSS2 were identified as the host factors for cleavage at this residue (31).

Aprotinin, purified from bovine lung (32), is a protease inhibitor with a long history of clinical use as an antifibrinolytic agent in cardiac surgery (33). Its potential as an anti-IAV drug has been recognized for over a decade (34) and has been shown to reduce the infectivity of a broad spectrum of IAV strains (34, 35) both *in vitro* (26) and *in vivo* (36). Once withdrawn from the Western drug market due to its association with mortality (33), aprotinin has been approved as a locally administered, small-particle aerosol drug for the treatment of IAV infection in Russia (36). However, side-effects associated with the systemic administration of aprotinin raises the need for an alternative protease inhibitor for use in treatment of IAV infections.

Camostat, a serine protease inhibitor, was reported to demonstrate anti-IAV potential in mice dating back to 1996 (37), but little to no research has been conducted to develop it into an anti-IAV treatment. It was revisited and proven to be one of the most efficient serine protease inhibitors for the inhibition of IAV replication in primary human tracheal epithelial cells *in vitro* when tested compounds were used at similar molarities (35). At present, camostat is widely administered for the treatment of liver fibrosis, chronic pancreatitis, and cancer (38, 39), making it a highly promising candidate for drug repurposing. Despite the lack of association between camostat and increased mortality (as with aprotinin), reports of camostat potentially inducing acute eosinophilic pneumonia (38) warrants the need for careful consideration and further research into the repositioning of drugs from the same class.

Highly pathogenic IAV, such as the H5 and H7 subtypes, are reported to have HA cleavage sites rich in basic residues (30). The polybasic nature of the cleavage sites provides multiple targets for a broad spectrum of proteases, including the more ubiquitously expressed intracellular proteases such as furin (40). This increased protease spectrum could be utilized by these viruses for the activation of HA prior to viral budding, allowing for evasion of potential inhibition by exogenously administered serine protease inhibitors. Furthermore, an *in vivo* study utilizing mice treated with a single protease inhibitor prior to infection with H7 virus bearing a polybasic cleavage site showed poor efficacy despite good results were obtained for infection with H1N1 virus bearing single cleavage site (41), suggesting strain specificity in using serine protease inhibitors to treat IAV infections.

#### Inhibition of Endosomal Acidification

Endosomal acidification is required for the release of IAV genome (in the form of a vRNP complex) into the cytoplasm (24). Research has shown that an increase in endosomal pH during the early phases of infection could inhibit IAV infection *in vitro* (42), bringing to light the possibility of controlling IAV infection through the prevention of endosomal acidification.

The V-ATPase inhibitor bafilomycin A1, when used at high concentrations (10–100 nM) has been proven to inhibit IAV replication through the efficient suppression of V-ATPase (43, 44). However, prominent cytotoxicity to host cells was also observed at such concentrations (44). Interestingly, lower concentrations (0.1 nM) of bafilomycin A1 lack inhibitory effects on V-ATPase attenuated IAV replication due to disruption of endosomal trafficking. Thus, bafilomycin A1 is suggested to exert its antiviral function *via* distinct mechanisms at differing concentrations.

Diphyllin, isolated from the plant *Cleistanthus collinus*, is a natural compound able to induce a V-ATPase inhibitory effect (45). In contrast to bafilomycin A1, diphyllin is well-tolerated *in vitro* without inducing obvious cytotoxic effects (46). Most notably, diphyllin is found to effectively inhibit replication of viral strains resistant to amantadine and/or oseltamivir (46). Since drug resistance to these widely administered antivirals is of major public health concern (13), diphyllin is regarded as a promising antiviral against drug-resistant IAV strains.

#### Controlling Cholesterol Homeostasis

The release of IAV genomic material during replication requires the fusion of the endosomal membrane with the viral envelope. Since cholesterol plays a major role in controlling the fluidity of the lipid bilayer in cells, it is hence suspected to have a role in the infection cycle of IAV.

Interferon-induced transmembrane proteins (IFITMs) are proteins expressed in many vertebrates (including humans) and are found on the plasma membrane, the membranes of early and late endosomes, as well as on lysosomes (47, 48). While humans express IFITM1, IFITM2, IFITM3, IFITM5, and IFITM10, only IFITM 1, 2, and 3 are both immune-related as well as interferon (IFN)-inducible (48), and have been observed to restrict the replication of different viruses, including IAV (49). Studies suggest that IFITMs limit viral infection by reducing membrane fluidity and hence restrict the hemifusion (the mixing of lipid bilayer without the release of viral content) of viral and endosomal membranes (50), probably *via* the disruption of cholesterol homeostasis of late endosomes, where viral fusion and genome release conventionally take place (51). A recent study using RNAi also demonstrated that cholesterol homeostasis can be regulated *via* acid phosphatase 2 (ACP2)-mediated Niemann–Pick C2 activity and impaired the membrane fusion of IAV and influenza B virus (IBV) (52), further suggesting the importance of controlling cholesterol homeostasis in the release of viral genome to cytoplasm.

On the contrary, later studies suggest that IFITM3 exerts its antiviral activity in a cholesterol-independent manner, showing that an increase in cholesterol composition of late endosomal membranes fail to inhibit viral membrane fusion (53). In addition, studies suggested the accumulation of cholesterol level in the late endosome does not inhibit the IAV genome release into cytoplasm (54, 55).

With the modulation of cholesterol levels in host endosomal membrane as a mean to inhibit IAV host cell entry is still under debate, further studies are required before clear conclusions can be drawn.

#### Other Possible Targets for IAV Entry Inhibition

By comparing the miRNA profiles of the IAV-permissive HEK 293T cells and (less permissive) HeLa cells, miRNA-33a has been identified as a negative regulator for IAV infection *via* the inhibition of archain 1 (ARCN1, also known as δ-COPI) (56). ARCN1 is a subunit of the COPI complex that is required for intracellular trafficking and endosome function (57), depletion of which has been reported to inhibit IAV infection (14). Despite impaired IAV internalization caused by ARCN1 depletion *via* siRNA (56, 58), it was not able to recapitulate through acute inhibition of COPI complex by pharmaceutical means (58). It is hypothesized that the long-term (lasting days) perturbation on ARCN1 by RNAi affected the general endosomal trafficking network, a phenomena which cannot be recapitulated by acute pharmaceutical inhibition to block IAV infection (58). The potential of targeting ARCN1 for IAV treatment deserves further investigation, despite the favorable results from RNAi studies.

### Blocking the Nuclear Import of vRNP Complex

Nuclear import of vRNP complexes from the cytoplasm following fusion of the viral and the endosomal membrane is required for replication to take place (59). An early study suggested that vRNP complexes could be transported to the periphery of the nucleus (60), while recent studies report that vRNP complexes utilize the importin-α-importin-β1 (IMPα-IMPβ1) system for nuclear import (59, 61) and lacking of importin-α7, in an importin-α7 knockout mouse model were found to be resistant to IAV infection (62).

Ivermectin has long been clinically administered for the treatment of parasitosis (63), but has recently come to attention as a potential inhibitor of IMPα/β (64). Ivermectin inhibition of IMPα/β has shown to inhibit the replication of RNA viruses such as dengue virus and HIV-1 (64). Ivermectin was recently tested for the inhibition of IAV *in vitro*, with nuclear import of vRNP complex (of both wild-type and antiviral MxA escape mutant) efficiently inhibited (65). Given ivermectin’s longstanding record of clinical applications and FDA-approved status, repurposing of this drug for the treatment of IAV should be considered, especially while under threat of pandemic IAV outbreak.

### Genomic Replication and Protein Synthesis

Following the import of the vRNP complex into the nucleus of the host cell, RdRP uses the vRNA as a template to synthesize mRNA or cRNA. Synthesized cRNA remains in the nucleus for new vRNA generation, while mRNA is exported out of the nucleus for translation. Viral protein products are either transported to the cell surface *via* Golgi (in case of HA and NA) or imported back into the nucleus to bind with vRNA, forming new vRNP complex (59). Numerous host factors are involved in this process and hence could be possible targets for therapeutic intervention.

#### Regulation of the Splicing of Pre-mRNA

Out of the eight genome segments of IAV, the M and NS segments are well known for undergoing splicing to generate at least two different mRNAs per individual segment (66, 67). Cdc2-like kinase 1 (CLK1) is a kinase which regulates alternative splicing of pre-mRNA (68). Inhibition of CLK1 by the chemical TG003 or knockdown of CLK1 is shown to cause a decrease in M2 mRNA generation and disrupt downstream M2 protein expression, prominently reduced IAV propagation (15).

Clypearin and corilagin were both found to be potent anti-IAV compounds, with a higher therapeutic index than TG003 *in vitro* (69). Clypearin is isolated from herbs used by Chinese medicine practitioners for treating respiratory tract diseases (69), while corilagin is isolated from medicinal plants and herbs. The identification of effective compounds and the systemic investigation of the use of traditional Chinese medicine (TCM) in the treatment of IAV infection open new frontiers in research and therapeutics.

#### Inhibition of mRNA Export

During replication, viral mRNA is exported from the nucleus to cytoplasm, where protein synthesis takes place. Human RNA polymerase II activity is found to be correlated with IAV replication through the inhibition of nuclear export of certain viral mRNAs, such as M1 mRNA (70).

Cyclosporine A (CsA) is a FDA-approved drug with immunomodulatory functions (71) that has been found to have an anti-IAV effect in both cyclophilin A (CypA)-dependent and -independent manners (72). The CypA-dependent effect was found to correlate with nuclear export of vRNP complex (see Targeting Nuclear Export Complex). The CypA-independent effect caused inhibition of host RNA polymerase II. CsA is a prospective drug candidate for treatment of IAV infections with a relatively high barrier for development of intrinsic drug resistance, as opposed to commonly used antivirals (73).

Nuclear RNA export factor 1 (NXF1) is a host factor that has been identified to be involved in the nuclear export of IAV mRNA. The knockdown of NXF1 in HEK 293T cells revealed prominent viral mRNA nuclear retention in host cell nucleus (74). Protectin D1 (PD1), an endogenously produced lipid in the respiratory tract, has been identified to have potent anti-inflammatory and antiviral effects (75). PD1 production was notably found to be reduced in the lungs of IAV-infected mice. Therapeutic administration of PD1 was shown to significantly reduce IAV mRNA expression, lower lung viral titer, as well as improve survival of IAV-infected mice. Mechanistic studies revealed attenuated cytoplasmic translocation of viral mRNA with such treatment. A decrease in recruitment of viral transcripts to NXF1 was observed while nuclear export of host RNA remained largely unaffected, suggesting a role of PD1 in regulating NXF1 in nuclear export of viral RNA. Natural PD1 expression in the human airway makes this an ideal candidate for novel therapeutics in the treatment of IAV infection.

#### Inhibition of mRNA Translation

The eukaryotic initiation factor-4A (eIF4A) family plays an important role in protein translation (76, 77). eIF4A impairment has been proven to be related to antiviral activity in a broad spectrum of RNA viruses *in vitro* (78), with inhibition of IAV mRNA translation (79). The eIF4A inhibitors, silvestrol and pateamine A were demonstrated to arrest viral protein synthesis, thus blocking viral genome replication *in vitro* (80). Although both silvestrol and pateamine A caused high cytotoxicity at the concentration required effective for IAV inhibition, drugs targeting mRNA translation for various diseases have been approved by FDA or are under active development (81). As such, inhibition of IAV infections by disrupting mRNA translation may well be a therapeutic approach in the future.

#### Inhibition of HA Maturation

Post-translational modifications during protein maturation ensure proper function of proteins, with proteins of IAV no exception. Nitazoxanide, a FDA-licensed drug used to treat enteritis, was found to be effective in controlling IAV infection by interfering with HA N-glycosylation as well as intracellular trafficking in host cell and eventually led to a reduction in viral budding (82). Despite the mechanism of nitazoxanide being presently unknown, its ability to inhibit replication of numerous viruses [IAV, respiratory syncytial virus, coronavirus, hepatitis B virus, and many others (83)] suggests that it may act on host machinery. The drug has also been proven *in vitro* to inhibit the propagation of many circulating strains of human IAV, including those resistant to oseltamivir or zanamivir (84). Nitazoxanide has a high barrier of resistance to IAV (83) and other viral strains resistance to neuraminidase inhibitors (85), making it a very promising therapeutic target for IAV treatment. The drug is currently under phase III clinical trials (83).

### Nuclear Export, Assembly, Apical Transport, and Viral Budding

In the later stage of viral replication, viral RNAs of IAV packed with RdRP and NP (known as vRNP complexes) are exported from the nucleus (59), assembled (86), and transported to the plasma membrane [apical in polarized cells (87)] for budding.

#### Inhibition of Nuclear Export

##### Targeting Nuclear Export Complex

Exportin 1 (XPO1, also known as CRM1) is well known for its function in the nuclear export of protein (88) and RNA, including viral RNA (89). Similar to HIV (89, 90), IAV viral RNA does not directly bind to XPO1 but is instead held together by several viral proteins. The viral nuclear export protein (NEP, or previously known as NS2) and the vRNP complex have been proposed as the nuclear export complex (91). Cellular XPO1 has been proven to be crucial in the nuclear export of the vRNP complex, with early studies using leptomycin B (LMB), a potent XPO1 inhibitor, revealing that *in vitro* inhibition of XPO1 led to nuclear retention of vRNP complex (92, 93). However, LMB was deemed unsuitable for development as a potential drug in the phase I clinical trial due to observed cytotoxic effects (94).

Verdinexor (also known as KPT-355) is a new bioavailable selective inhibitor of XPO1. It has been shown to be effective against different strains of IAVs both *in vitro* and *in vivo* as prophylactic and therapeutic treatments (95, 96). It is worth mentioning that delayed administration of verdinexor at day 4 post-infection was still deemed beneficial, with reduced viral load *in vivo* (96). This suggests a prolonged therapeutic time window when compared to the mainstay antiviral drugs such as oseltamivir, where recommended administration is at the early stage of infection (within 48 h of symptom onset) (97). Currently, verdinexor has passed the phase I clinical study trials, suggesting that it does not pose severe cytotoxic effects as LMB does.

In addition, a recent report demonstrated that a new drug, DP2392-E10, which binds and inhibits the function of XPO1, can suppress IAV replication *in vitro* (98) further strengthens the concept of IAV intervention by targeting XPO1.

Viral M1 protein is crucial in assisting the nuclear export of vRNP complex. It was commonly suggested that M1 protein links vRNP complex to viral nuclear export protein NEP which interacts with XPO1 for nuclear export (59). Thus, viral M1 protein may serve as a target to inhibit nuclear export of vRNP. As previously mentioned (see Inhibition of mRNA Export), CsA inhibits IAV replication *via* both CypA-dependent and -independent mechanisms. A recent study using a transgenic mice over-expressing CypA showed greater resistance to IAV challenge (99). In the CypA-dependent mechanism, CsA enhances the binding of CypA to M1 protein (72), increases the self-association of M1, and hinders M1 nuclear import (100). CsA also promotes the CypA-dependent degradation of viral M1 protein (72, 101). CsA seems to be a promising drug to inhibit the nuclear export of vRNP complex by inhibiting viral M1 protein stability and function.

Recently, CD151, a tetraspanin (defined by four transmembrane domains with conserved residues) that is expressed abundantly in lungs and interacts with integrins has been implicated in the regulation of IAV replication *in vitro* and *in vivo* (102). Knockdown of CD151 in primary human nasal epithelial cells resulted in the nuclear retention of host XPO1, viral NP, NEP, and M1 proteins, with an increased survival rate observed in IAV-infected CD151 knockout mice. Co-immunoprecipitation assays suggest that CD151 interacts with viral NP, M1, and NEP proteins (102); however, the exact domains involved in interaction and the mechanism of CD151 function in nuclear export remain unclear. Given that a small molecule inhibitor for CD151 is now under development (103), more data revealing the role of CD151 in IAV infection and subsequent use in targeting CD151 as anti-IAV therapy is anticipated.

##### Targeting the Raf/MEK/ERK Pathway

During IAV infection, Raf/MEK/ERK signaling cascade is activated, while the inhibition of MEK by U0126, probably mediated *via* myosin (light chain) (104), a known motor protein, impairs the nuclear export of vRNP complexes (105). Suppressing IAV replication by inhibition of Raf/MEK/ERK signaling cascade has been illustrated both *in vivo* (106) and *in vitro* (105). The replication of IBV (107) as well as Borna disease virus (108) was shown to be inhibited by U0126, suggesting the versatility of this approach in controlling infection by different viruses. Despite being effective when administered locally to lungs *via* aerosol, U0126 has little effect when administered orally (106).

Another MEK inhibitor, CI-1040 (also known as PD184352) was shown to have high potency against IAV *in vitro* (106). CI-1040 has completed phase II clinical trials as an anti-tumor drug, with the application of CI-1040 as a potential anti-IAV drug candidate recently revisited. Unlike U0126, CI-1040 is orally bioavailable and oral administration of CI-1040 at 48 h post-infection protected 60% of the IAV-infected mice, while the oseltamivir-treated group experienced a 100% death rate (109). Oseltamivir is known to be effective only when administered in the early stages of IAV infection. This suggests the potential use of CI-1040 as an agent used in IAV treatment due to its potentially longer therapeutic time window than mainstay antivirals.

Formyl peptide receptor 2 (FPR2) located at the host cell surface was identified as an ERK stimulator (110). Antagonizing FPR2 promoted the survival of IAV-infected mice (110). Furthermore, FPR2 antagonists have been described to possess antiviral activity against not only IAV but also IBV infection (111), promoting the idea that antagonizing FPR2 to suppress Raf/MEK/ERK signaling cascade could potentially be a novel approach for the treatment of a broad spectrum of influenza viruses.

#### Apical Transport of Viral Components

After the nuclear export of the vRNP complexes, host cell’s intracellular transport mechanism is required to deliver vRNP complexes to the host plasma membrane for the assembly of viral RNAs and proteins at the final stage of viral replication. Among the various vesicular compartments found in a cell, the Rab11A^+^ endosomes are known to recycle endocytosed membrane proteins and lipids to the plasma membrane for membrane homeostasis (112), a property utilized by many RNA viruses, including IAV (87, 113–115). IAV progeny virus production was found to be significantly reduced in Rab11A^+^ knockdown human cell lines (116). Furthermore, vRNP complex plasma membrane transport perturbation was observed in *Rab11A* knockdown cells (114, 115); in cells expressing deletion mutant of Rab11 family interacting proteins (87); as well as cells treated with chemicals to interfere microtubule (114). Direct interaction of vRNP complex with Rab11A has also been verified (114, 115), demonstrating the dependence of vRNA complex transport on Rab11A^+^ vesicles and the microtubule network during viral replication. Since Rab11A proteins do not confer any mobile properties to the vesicle, molecular motors such as kinesins are required for the active transportation of vesicles through cytoskeletons.

KIF13A, a kinesin-3 family member, was recently identified as a molecular motor for plasma membrane transportation of vRNP-loaded Rab11A^+^ vesicles (117). KIF13A knockdown was found to reduce progeny virus production. Overexpression of a mutant form of KIF13A lacking in motor capacity resulted in disruption of the plasma membrane distribution of vRNP complex during later stages of infection. This data suggest that the apical transport of viral components *via* Rab11A or KIF13A could potentially serve as therapeutic targets against IAV infection. Further examination is merited.

Tubulin acetylation and deacetylation affects microtubule stability (118). Histone deacetylase 6 (HDAC6) was found to deacetylate α-tubulin, one of the subunits of microtubule (119). A study has demonstrated that HDAC6 is involved in IAV replication (120). Inhibition of HDAC6 by tubacin or knockdown of HDAC6 gene resulted in an increase of progeny virus production with vRNP complex redistributed toward the periphery of infected cells. In addition, transportation of HA to the plasma membrane for viral budding was also found to be inhibited by HDAC6. This data suggests that activation of HDAC6 by its stimulant could be a potential approach to anti-IAV therapy, despite HDAC6 stimulants still being under development.

#### Interference of Viral Budding

While several studies have suggested IAV transmission between cells through apical membranes (121) and intercellular connections (122), virus budding from cell membranes remains the major route for transmission of viruses to uninfected cells. NA is responsible for the cleavage of sialic acid to prevent the interaction between HA and the host cell during viral budding. Besides, viral NA, viral HA, M1 as well as M2, are also suggested to play an important role in the initiation of the budding process (123, 124).

In Section “Controlling Cholesterol Homeostasis,” we discussed the involvement of host cholesterol in viral membrane fusion and viral genome release to cytoplasm. Recent studies have demonstrated that host cholesterol may also play an important role in viral budding. It was demonstrated that overexpression of annexin A6 (AnxA6), a phospholipid binding protein, could lead to a decrease in cholesterol levels within the Golgi apparatus and plasma membrane (55), ultimately causing a reduction in egression of progeny virion from infected cells (54). This reduction could be reversed by the addition of exogenous cholesterol (55). Similar to AnxA6 overexpression, addition of a hydrophobic polyamine, U18666A, could reduce cholesterol level in plasma membrane, also inhibited viral replication (55). Since IAV is assumed to bud from lipid rafts (cholesterol-rich plasma membrane domains) (123), it was demonstrated that AnxA6 overexpression or U18666A treatment could hinder progeny virus production by lowering the cholesterol content in the plasma membrane. This hypothesis was strengthened through recent studies resolving the cholesterol-binding site of viral M2 protein, suggesting that IAV M2 clustering (which provides membrane curvature for scission) is mediated by cholesterol (125). A recent report utilizing two different FDA-approved cholesterol-lowering drugs, gemfibrozil and lovastatin, stated that there was reduction in stability and infectivity of progeny virus compared to that replicating within cholesterol-sufficient host cells (126). Taken together, this data suggests that controlling cellular cholesterol content would be an effective alternative with drugs available for repurposing IAV treatment. Further *in vivo* works are needed to confirm this hypothesis.

The Gi-type G-protein coupled receptor α2-adrenergic receptors (α2-ARs) have been recently identified as a key host factor involved in IAV replication (127). Apical transport of the viral protein HA is inhibited by low intracellular cAMP level after stimulating the α2-AR-mediated signaling. *In vitro* stimulation of α2-AR by its agonist clonidine inhibits IAV replication. Therapeutic administration of clonidine reduced pulmonary edema and improved survival rate of IAV-infected mice. Development of a new antiviral targeting the α2-AR-mediated signaling seems promising and deserves further investigation.

### Interrupting the Virus Replication Cycle by Combinatory Use Targeting Both Virus and Host Factors

Although targeting host factors for viral interventions generally provides a better resistance barrier, emergence of resistance may still arise (61). Therefore, combined use of interventions targeting both virus and host factors have been recommended to reduce opportunities for viral development of resistance. One such example would be the combined administration of NA inhibitor (oseltamivir) alongside an anti-host factor [such as V-ATPase inhibitor diphyllin (46), HA maturation inhibitor nitazoxanide (85), FPR2 antagonists (111), and XPO1 inhibitor verdinexor (96)]. While further direct assessment for the ease of emergence of escape mutants between single and combinatory use of drugs is required, the synergistic effects of a combined, multi-drug approach observed thus far highly suggest an increased effectiveness over a single-drug approach.

Table 1 summarizes novel host targets regulating IAV replication. Compared to RNAi, small molecular chemicals remain the best choice as drug candidates due to their fast acting and easy-to-deliver properties. Although small molecular chemicals targeting certain host factors aforementioned have yet to be developed, their RNAi-identified involvement in the IAV replication cycle provide leads for the development of new IAV interventions.

**Table 1:** Advancements on targeting host factors for antivirals.

Cellular target	New potential therapeutic approach	Suggested function	Reference
**Viral entry**			
Serine proteases	Camostat	Inhibits HA0 cleavage	Yamaya et al. (35)
V-ATPase	Diphyllin	Inhibits endosomal acidification	Chen et al. (46)
Acid phosphatase 2	siRNA	Indirectly disrupts cholesterol homeostasis	Lee et al. (52)
**Nuclear import**			
Importins	Ivermectin	Inhibits nuclear import of viral ribonucleoprotein (vRNP) complex	Gotz et al. (65)
**Genomic replication and protein synthesis**			
Cdc2-like kinase 1	Clypearin	Attenuates M2 splicing	Zu et al. (69)
RNA polymerase II	Cyclosporine A	Inhibits viral mRNA export	Ma et al. (73)
Nuclear RNA export factor 1	PD1	Inhibits viral RNA export	Morita et al. (75)
Eukaryotic initiation factor-4A	a. Silvestrol b. Pateamine A	Inhibits mRNA translation	Slaine et al. (80)
**vRNP complex nuclear export**			
XPO1	a. Verdinexor b. DP2392-E10	Inhibit vRNP complex nuclear export	a. Perwitasari et al. (95, 96) b. Chutiwitoonchai et al. (98)
CypA	Cyclosporine A	Promotes M1 degradation	Liu et al. (72, 101)
CD151	siRNA	Inhibits vRNP complex nuclear export	Qiao Y. et al. (102)
MEK	CI-1040	Inhibits MEK to suppress phosphorylation of myosin light chain leading to nuclear retention of vRNP complex	Haasbach et al. (109)
Formyl peptide receptor 2 (FPR2)	WRW4	Inhibits activation of Raf/MEK/ERK by the ligation of AnxA1 to FPR2	Courtin et al. (111)
**Viral component apical transport**			
KIF13A	To be determined	Blocks vRNP apical transport	Ramos-Nascimento et al. (117)
Histone deacetylase 6	To be determined	Regulates microtubule stability	Husain et al. (120)
AnxA6	To be determined	Reduces plasma membrane cholesterol level and decreases virion egress or stability	Musiol et al. (55)
Cholesterol	U18666A	Reduces plasma membrane cholesterol level and decreases virion egress	Musiol et al. (55)
α2-adrenergic receptors	Clonidine	Reduces intracellular cAMP to impair Influenza A virus HA plasma membrane transport	Matsui et al. (127)
**Other**			
Cholesterol	a. Gemfibrozil b. Iovastatin	Reduces progeny virus stability and infectivity	Bajimaya et al. (126)

## Regulation of Aberrant Immune Responses in IAV Infection

The immune system aims to protect the host from infection and clear the pathogen once an infection occurs. In addition, the complex networks formed between the host physiology and the immune system co-operatively shape the disease outcome; modulations on the networks could alleviate disease severity in IAV infections.

The immunological responses elicited by IAV infection has been reviewed in detail (128–130). At the initial stage of IAV infection, the respiratory epithelial cells are the primary target for infection. Once the infection is initiated, the recognition of infection is accomplished *via* the detection of pathogen-associated molecular patterns (PAMPs) by pattern recognition receptors (PRRs) (see Toll-Like Receptors), and lead to the expression and secretion of different cytokines and chemokines, such as IL-6, IL-8, tumor necrosis factor (TNF)-α, and CCL2 as well as type I and III IFNs. As sentinel cells, alveolar macrophages could also be infected, inducing cytokines and is the main source of type I IFNs (128, 129). Type I IFNs are known inducer for the upregulation of death receptor 5, which is the receptor for TNF-related apoptosis-inducing ligand (TRAIL), in lung pneumocytes (128). IL-8 and CCL2 produced by both epithelial cells and macrophages act as chemoattractants for neutrophils and monocytes, respectively. Neutrophils are one of the earliest immune cells being recruited to the site of infection (131) with transmigration of neutrophils carry out by adhesion molecules, such as CD11a, CD11b, and CD18 (132). In addition to the antiviral activity of neutrophil-released reactive oxygen species (ROS), defensin and pentraxin (132), uptaking IAV by neutrophils could also help in controlling viral propagation as these cells do not support replication of IAV (133). Besides controlling viral replication, neutrophils also play an important role in guiding the migration of IAV-specific CD8^+^ T-cells in the infection site by secreting and leaving a trail of CXCL12 (134). Infiltrated monocytes will, however, differentiate into macrophages or dendritic cells (DCs). The monocytes-derived macrophages are reported to be a permissive host for IAV production (135), sustaining inflammation by producing cytokines in a magnitude larger than that of the resident alveolar macrophages. The monocyte-derived DC as well as the resident airway CD11c^low^B220^+^ plasmacytoid DC (pDC) and two types of conventional DCs (CD103^+^CD11b^low^ and CD103^−^CD11b^hi^) acquire the antigen of the invading pathogen through either direct infection or up-taking infected dead cells (129). In the presence of type I IFNs, DCs mature when encountering PAMPs from invading pathogen (129). Depending on the sub-cellular localization of the antigen, cytosolic and endosomal antigen will be loaded onto major histocompatibility complex (MHC) class I and II molecules respectively (130). Once mature, DCs migrate from the infection site to the draining lymph nodes *via* the interaction of CCR7 and CCL19/CCL21 (130, 136) for antigen presentation *via* MHC class I and II to naïve CD8^+^ and CD4^+^ T-cells, respectively (137–140). Interestingly, monocytes-derived DCs that engulfed the infected dead cells are poor antigen presenters for CD8^+^ T-cells and require the transfer of intact MHC class I/peptide complex to lymph node-resident CD8α^+^ DCs which are the most efficient antigen-presenting cells to CD8^+^ T-cells (137). In addition to antigen presentation, pDC are well known for their high ability in type I IFNs production to limit viral propagation (141).

**Figure 1:**
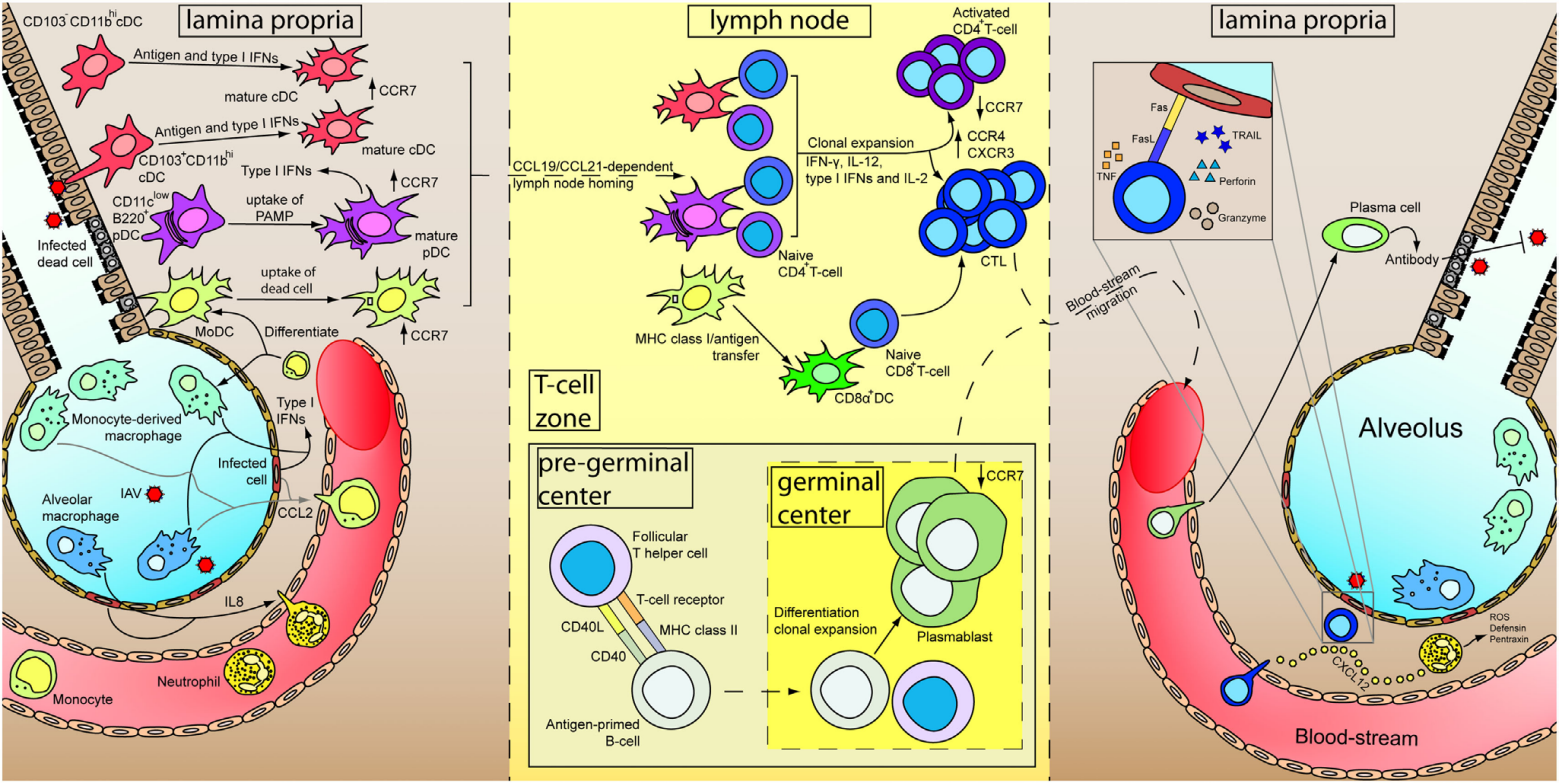
Immune responses of primary influenza A virus (IAV) infection. At the initial stage of infection (left), airway epithelial cells and pneumocytes are major targets for IAV. Various cytokines are secreted by these cells as well as alveolar macrophages to induce inflammation and recruits blood immune cells. Infiltrated neutrophils secrete different antimicrobial products to control viral replication. Besides, CXCL12 is secreted by neutrophils to guide the cytotoxic T-lymphocytes (CTLs) in the later stage of infection. Infiltrated monocytes will further differentiate into monocyte-derived macrophages and monocyte-derived dendritic cells (MoDC). Constant surveying of the airway and uptake of virus-infected dead cells by DCs lead to their maturation. Upregulation of CCR7 results in a CCL19/CCL21-dependent lymph node homing of DCs. Within the lymph node (middle), MoDCs cross-dress CD8α^+^ DC. CD4^+^ and CD8^+^ T-cells are activated by DCs in the presence of cytokines and undergo clonal expansion. Antigen-primed B-cells mature with the aid from follicular T helper cells and further differentiate into plasmablasts in the germinal center. Differential expression of receptors upon maturation of T- and B-cells prompt them to leave the lymph node, enter the blood-stream and recruited to the lung. In the inflamed lung (right), neutrophils leave a trail of CXCL12 to guide CTLs migration. Measures utilized by CTLs for killing infected cells are depicted in inset. Plasmablasts further terminally differentiate into plasma cells and increase antibody production for IAV neutralization.

Within the lymph node, naïve CD8^+^ T-cells are activated by the DCs, differentiate and clonal expand into cytotoxic T-lymphocytes (CTLs) with the aid of various cytokines, including IFN-γ, IL-12, type I IFNs, and IL2 (142, 143), and the help from activated CD4^+^ T helper cells (144). Differentiated CTLs downregulate their lymph node homing receptor CCR7 and upregulate CCR4 and CXCR3 for the migration to the site of infection. Within the site of infection, CTLs control viral replication by targeting and inducing apoptosis of virus-infected cells *via* the secretion of perforin and granzymes as well as the ligation of death receptors on the infected cells by TNF, Fas ligand, and TRAIL. On the other hand, CD4^+^ T-cells are activated by the presentation of MHC class II/antigen complex by DCs, with co-stimulatory receptors such as CD28 expressed on the T-cells and the ligand for CD28 (CD80 and CD86) expressed on DCs playing an important role (144). Activation of CD4^+^ T-cells lead to differentiation into different effector cells subsets, including the classical Th1 and Th2, and the more recently identified regulatory T cells, follicular T helper cells, Th9, and Th17 subsets (144). Th1 cells regulate to the differentiation of CTLs as mentioned whereas Th2 cells contributes to the activation of B-cells through CD40L. Within the pregerminal center of the lymph node, the follicular T helper cells interact with antigen-primed B-cells and promote their proliferation. Antigen-primed B-cells differentiates into plasmablast and undergo antibody class-switching in the germinal center (145). Detailed functions of regulatory T cells, follicular T cells, Th9, and Th17 cells are discussed elsewhere (144, 145). Plasmablasts enter the blood-stream, are recruited to the inflamed tissue, and terminally differentiate into plasma B cells which specialize in the production of antibody for pathogen neutralization, opsonization, and antibody-dependent cell-mediated cytotoxicity, etc. Memory T- and B-cells are also developed during the maturation process, and has been discussed and reviewed elsewhere (146–149). A schematic diagram showing a summary of the immune response after IAV infection has been illustrated in Figure 1.

The Yin and Yang theory is always used to describe the importance in balancing the host immune response. In the light of this theory, the treatment strategy aims to suppress the overwhelming activation of the host immune response and in reverse to compensate any unfavorable suppression.

Although adaptive immune responses are important in viral clearance, the immediate innate immunity play an important role in the early control of an infection, and conversely, is a major factor for disease severity due to immunopathology. Dysregulated immune responses caused by viral infections have been implicated in severe disease development (150, 151), such as acute lung injury (ALI). ALI in its most severe form, known as acute respiratory distress syndrome (ARDS), is reported to be the most prevalent cause of mortality in IAV-infected patients (152).

Studies suggested that IAV strains could be associated with either over-activating (human infection by avian H5N1 and H7N9) (153, 154) or suppressing (H1N1, H3N2) (155) immune response.

### Regulation of Neutrophil Infiltration and Neutrophil Extracellular Trap

Recent history has seen the outbreak of IAV pandemics of varying severity takes place at the cost of millions of lives. One such example would be the deadly Spanish flu of 1918, which claimed the lives of 20–50 million of the 500 million people infected worldwide.

The pathological examination of lung sections from mice infected with reconstituted 1918 IAV virus revealed necrotizing bronchiolitis and severe alveolitis in tissue, with neutrophils observed as the predominant inflammatory cell type present (156), suggesting neutrophil involvement in the pathogenesis of IAV infection.

The majority of immune cells in blood circulation are neutrophils; of which they are among the first innate immune cells recruited to the site of infection (131). Neutrophils characteristically control microbial infections by generating bactericidal (157) neutrophil extracellular traps (NETs), consisting of granule proteins, histones, and decondensed chromatin (131). Both protective and destructive role of neutrophils in IAV infections have been described. The contrasting role of neutrophils could be explained by factors such as viral strain and viral dose used in different experimental setup, etc.

The protective role of neutrophils was observed when mice infected with a low, non-lethal dose of IAV H3N2 strain HKx31 displayed neutrophil-mediated viral clearance *via* phagocytosis (132, 158). Depletion of neutrophils has found to enhance viral load in the IAV-infected animals (158).

On the contrary, this protective nature is disputed due to the association of neutrophil-generated NETs. Extensive NET formation was observed in mice infected with PR8, an IAV strain highly pathogenic to mice (159). Histones and myeloperoxidase within the NET induce cell death of lung epithelium and endothelium (157), leading to the loss of integrity of the alveolar-capillary barrier, a characteristic of ALI. Yet, while histones have been shown to suppress IAV replication *in vitro* (160), *in vivo* study demonstrated that there was increase in lung inflammation and damage in IAV-infected mice treated with histones (161). Interestingly, co-treatment of lethally infected mice with anti-histone antibody and oseltamivir resulted in an increase in animal survival when compared to infected mice groups treated solely with oseltamivir (161).

In agreement with the *in vitro* and *in vivo* data, it has been reported that NET produced by cultured neutrophils from patient with H7N9 and severe H1N1 infection increased alveolar epithelial cell permeability (162) leading to ALI. More importantly, plasma NET level positively correlated with the disease severity index (including higher acute physiology and chronic health evaluation II score) and multiple organ dysfunction syndrome (162), further demonstrating the detrimental role of NET in the pathogenesis of severe IAV infections.

Studies have demonstrated the involvement of superoxide dismutase and myeloperoxidase in NETosis, the formation of NET (159). The presence of anti-myeloperoxidase antibody as well as the superoxide dismutase inhibitor (DETC) significantly reduced NETosis. Finally, tetrahydroisoquinolines (163) and a pan-peptidylarginine deiminase (PAD) inhibitor, named Cl-amidine (164) have been suggested to inhibit NETosis. Despite it has been reported that during IAV H1N1 infection, PAD4 knockout mice displayed only slight improvement in weight loss and a slight prolonged but no end-point survival advantage was observed compared to WT mice (165), based on the extensive findings presented above, targeting NET to prevent ALI in the severe case of IAV infection, including the highly pathogenic avian IAV, remain promising and may warrant further investigation.

### Innate Lymphoid Cells (ILCs)

Innate lymphoid cells are cells of lymphoid lineages that do not express antigen-specific B- or T-cell receptors (166). Similar to T-helper cells, they are classified into subsets by their ability to produce type 1 (Th1), type 2 (Th2), and type 3 (Th17 and Th22) cytokines.

Previous studies confirmed the involvement of ILCs of group 2 linage (ILC2) in IAV infection and airway inflammation (166, 167). On the positive side, during the recovery phase of IAV infection, ILC2 expresses amphiregulin which promote airway epithelium repair (166, 168), thus facilitating the recovery of the infected lung.

On the other hand, in response to IL-33 produced by macrophages, DCs, and NKT cells, ILC2 secretes IL-5 and IL-13 and induce airway hyper-responsiveness. Recruitment of eosinophils by IL-5 to the lung also mediates airway inflammation (166). Since eosinophilia is a characteristic of allergic asthma and influenza is a major cause for morbidity and mortality in asthma patients (166), it will be of particular interest to investigate the role of ILC2 in IAV infection, particularly in asthma patients.

ILC1s have been initially described as immature NK cells residing in the liver and share many phenotypic similarities with NK cells (169). It was recently appreciated that tissue-resident ILC1s other than the previously recognized NK cells are the major early source of the antiviral IFN-γ at the primary site of various viral infection, including IAV (170). Interestingly, IFN-γ was found to suppress ILC2 activity and reduce IL5 production which exacerbates disease severity during influenza A(H1N1)pdm09 infection (171). This data may highlight a link between ILC1 and ILC2 and suggesting ILC1 can suppress ILC2 activity *via* IFN-γ production during IAV infection.

With ILCs finally identified, functions of these cells and their role in immune response to tumors and pathogen infections have been massively investigated in recent years. Type I IFNs, prostaglandin I_2_, corticosteroids, and testosterone have been reported to suppress ILC2 activity (172, 173). In addition to IL-33, the epithelial cytokines IL-25, thymic stromal lymphopoietin, as well as the lipid mediator prostaglandin D_2_ were found to activate ILC2 (173). The therapeutic potential of these ILC2 activators and suppressors is yet to be deduced. With more and more studies demonstrating the involvement of ILC in IAV infection, the interplay between different ILC subtypes in IAV infection would, therefore, be an interesting area to explore and modulate the ILC activity may be a future approach to combat IAV infection.

### Reactive Oxygen Species

Reactive oxygen species, generated by specialized enzymes such as NADPH oxidases, are released during IAV infection (174). The NADPH oxidase family consists of enzymes containing different catalytic subunit named Nox1–5 and dual oxidase (Duox) 1 and 2. ROS have been reported to display both beneficial (limiting viral replication) and detrimental (promoting ALI) effects in the course of IAV infection. Interestingly, the protective or destructive effect of ROS is dependent on the enzyme of which the ROS is generated (174).

Dual oxidase1 and 2 are found to be host-protective (174, 175). *In vitro*, ROS generated by nuclear Duox indirectly regulates the splicing of IAV mRNAs *via* the nuclear speckle-associated splicing complex (175). In addition to altering viral mRNA splicing, ROS generated by Doux2 has been attributed to the production of IFN-λ, an important anti-IAV IFN. In response to IAV infection, increased viral mRNA replication was observed when *Duox2* was silenced *in vitro* (176). Increased viral replication was also observed in mice with *Doux* silenced (175), further depicting the protective role of Doux in IAV infection.

Unlike Doux, Nox2 activation could be harmful to host. IAV infection was reported to induce Nox2-dependent endosomal ROS production (177). ROS could target the conserved Cys98 on Toll-like receptor (TLR) 7, and inhibit TLR7-mediated type I IFN expression during a mild IAV H3N2 infection *in vivo* (177). IAV-infected mice treated with specific Nox2 inhibitor, cholestanol-conjugated gp91ds-TAT, were found to have reduction in endosomal ROS production, restored TLR7 activity, and displayed a decreased viral load (177). In addition to Nox2, Nox4-dependent ROS production has also been reported to activate MAPK/ERK signaling (178), enhancing the export of vRNP complex, thus increasing viral replication (see Targeting the Raf/MEK/ERK Pathway). *Nox4* knockdown resulted in a reduction of viral replication *in vitro* (178).

Targeting the different NADPH oxidase isoforms, instead of scavenging ROS should be considered as the therapeutic approach for IAV infection, as Doux-mediated ROS production is beneficial (175, 176), while Nox2 and Nox4 are harmful during IAV infections (177, 178). Finally, NS1 (not to be confused with IAV NS1 protein) has been demonstrated to be a Nox inhibitor, which could inhibit the activity of Nox1, Nox2, and Nox4. A study demonstrated that NS1 suppresses IAV-induced Nox2 and significantly inhibits IAV virus replication (179). Besides cholestanol-conjugated gp91ds-TAT and NS1 aforementioned, apocynin, a phagocytic Nox2 inhibitor as well as ROS scavenger (180–182), has been demonstrated to ameliorate hyper upregulation of cytokines induced by IAV infection through SOCS1 and SOCS3 *in vitro* (154) and reduce peri-bronchial inflammation and viral titer *in vivo* (183). Interestingly, ebselen, another Nox2 inhibitor and glutathione peroxidase mimetic, could reduce inflammatory status measured in bronchoalveolar lavage fluid (BALF) of mice pre-exposed to cigarette smoke and subsequently infected with IAV (184). Taken together, these reports highlight the potential use of NADPH oxidases inhibitors and ROS scavengers to treat IAV infections.

### Soluble Mediators and Receptor-Based Immunomodulation

Dysregulated cytokine production has been associated with the elevated mortality rate observed in severe IAV infections (185, 186). As such, the immunomodulation of cytokines are regarded as promising therapeutic tactics. Recent advancements developed with this approach will be highlighted in the following section.

#### Tumor Necrosis Factor

Tumor necrosis factor has two main functions during viral infection—it activates NF-κB, inducing the expression of cytokines responsible for the host immune response; and induces apoptosis through activation of a signaling cascade involving TRADD, FADD, and caspase 3, 7, 8, and 10 (187–189). TNF is known to be highly upregulated in IAV-infected hosts, especially in hosts infected with highly pathogenic IAV (153, 190). However, it is both protective and counter-protective functions associated with TNF that makes it a target in the treatment of IAV.

The protective role of TNF is observed during infection by low pathogenic IAV, where extrinsically derived TNF is responsible for attenuating tissue-damaging CD8^+^ T-cell response (191). In addition to recruiting monocytic cells to the infection site, CD8^+^ T-cells response was observed to deteriorate lung pathology (192) and damage healthy, non-infected lung epithelial cells (193) upon IAV infection. Furthermore, TNF deficiency has been associated with an increased detection of IL-15 and IL-6 in BALF (192), which promote the survival of and proliferation of CD8^+^ T-cells (194, 195) and subsequent tissue damage. Exacerbated lung pathology caused by the upregulation of the monocyte chemoattractant protein-1 was observed in *TNF*^−/−^ mice infected with sub-lethal dose of IAV (196). In addition, decreased CD8^+^ T-cell contraction due to enhanced expression of the anti-apoptotic protein Bcl-2 was observed in sub-lethally IAV-infected TNF-deficient mice when compared to WT mice (192). As a whole, there is substantial evidence supporting the protective role of TNF in IAV infection.

On the other hand, the correlation of TNF with pulmonary edema has been well-documented (197). TNF has been observed to stimulate the expression of CXCL2 in alveolar epithelial cells in a transgenic mice model resembling extensive IAV infection in lung tissue, causing alveolar damage, lung edema, and hemorrhage (198). In addition to lung edema, TNF has also been reported to correlate with IAV-associated encephalopathy (199, 200). However, it is notable that despite IAV-associated encephalopathy, direct invasion of the central nervous system is rare (201), suggesting that IAV-associated encephalopathy could instead be a result of peripheral infection. Furthermore, TNF has been shown to increase the permeability of the blood–brain barrier (BBB) (202, 203), contributing to neural damage (204). These studies further support an anti-TNF approach as a potential therapy for severe IAV infection.

At present, etanercept, an anti-TNF drug administered in the treatment of rheumatoid arthritis, is the only TNF inhibitor (or even TNF directed treatment) tested for IAV treatment. Etanercept has been shown to protect against the *in vivo* lethal infection of mice with a highly virulent, mouse-adapted IAV strain (205), with observations made of an increased survival rate with decreased morbidity, expression of the proinflammatory cytokine IL-6, lung injury, and edema (205).

#### IL-6 and IL-27

The protective role of IL-6 was demonstrated in mice challenged with sub-lethal IAV infection. IL-6-deficient mice displayed exacerbated pulmonary damage (206, 207) and lung injury due to an observed decline in the survival of alveolar type II cells and alveolar epithelial cells (207). IAV suppresses the anti-apoptotic Mcl-1 and Bcl-X_L_ expression, causing cell death of neutrophils which are critical in viral clearance (206). Addition of IL-6 restored the expression of Mcl-1 and Bcl-X_L_ *in vitro* and is considered as the underlying mechanism for the observed survival advantage of WT mice over *IL-6* knockout mice during mild IAV infection.

IL-6 has also been shown to induce the proliferation of lung IL-10^+^ regulatory T cells and IL-27, which act to limit excessive proliferation of CD8^+^ T-cells and subsequent CD8^+^-inflicted damage. This would hence prevent the tissue damage observed in lung immunopathology (208).

Despite the apparent protective role of IL-6, high levels of IL-6 in serum or cerebrospinal fluid have been reported in severe neurologically complicated IAV cases, with IL-6 used as a marker for prognosis (199–201, 209, 210). The role of IL-6 in regulation of BBB permeability was reported (211), with potentially detrimental neurological complications. As such, the suppression of hyper-induced IL-6 as a form of therapy in severe IAV infection should be considered. One such option is the anti-IL6 antibody-based drug tocilizumab, which is currently administered clinically for the treatment of rheumatoid arthritis. However, study on the usage of this drug to treat hyper upregulation of IL-6 due to severe IAV infection has yet to be conducted. On the other hand, in a case of H1N1 virus-induced ARDS, the use of an extracorporeal cytokine hemoadsorption device to remove cytokines including TNF and IL-6 from the bloodstream (212) has showed beneficial to the patient (213). More research is required to confirm whether the removal or neutralization of IL-6 could be a potential therapy for severe IAV infections.

The activation of CD8^+^ T-cell is crucial for viral clearance. It should, however, be tightly regulated to limit CD8^+^ T-cell inflicted host cell damage. IL-6 mediates IL-27 induction (208). IL-27 acts to suppress CD8^+^ T-cells and reduce morbidity through IL-10 and regulatory T-cells (208). Much like other immunomodulatory approaches, the timing for applying IL-27 should be carefully assessed. Compared to placebo-treated IAV-infected group, early administration of IL-27 to IAV-infected mice in fact led to poorer viral clearance, increased morbidity, and deteriorated lung histopathology, while IL-27 administration during the recovery phase (5–10 days post-infection) accelerated recovery and improve lung immunopathology (214). Notably, IL-27 could also suppress Th17 responses and increases susceptibility to secondary *S. aureus* infection (215). Therefore, co-administration of antibiotics should be considered when utilizing IL-27 as potential IAV treatment.

#### Type I and III Interferons

Both type I and III IFNs have antiviral properties, with viruses counteract IFNs to gain an advantage for their propagation. The IAV viral protein NS1 inhibits the production of IFNs by antagonizing IRF-3, a key transcriptional factor for IFNs. This prevents the processing of cellular pre-mRNAs (including those for IFNs) and directly interacts with retinoic acid-inducible gene (RIG)-I receptors, which are critical in innate sensing, to suppress IFN production during infection (216, 217). In addition to inhibiting IFN expression, the induction of SOCS3 inhibits IFNs signaling by suppressing cytokine signaling has been documented (155).

The recognition of 5′ triphosphate on viral RNA by RIG-I receptor is shown to induce the expression of SOCS3, which in turn represses type I IFNs expression (155). Due to IFNs being a key contributor to antiviral immune response, an impairment of type I or III IFN production may cause the escalation of otherwise mildly pathogenic IAV infection into a life-threatening one (218).

While type I IFN has been demonstrated to inhibit IAV replication *in vitro* (219); the *in vivo* administration of type I IFN in animal models only displayed effectiveness in a prophylactic capacity. A lowered viral titer was detected in the nasal wash of test animals. However, host susceptibility to IAV infection remained unchanged (219). Notably, this protective effect is only conferred by an optimal dose of type I IFN of low to moderate amounts (10–100 units per mice daily); with higher dosages (1,000–10,000 units per mice daily) shown to increase morbidity (220). In addition, clinical trials demonstrated that prophylactic administration of type I IFN reduced disease severity and lowered susceptibility to IAV in males and participants aged 50 or above (221).

Despite relatively successful results seen in the prophylactic use of IFNs, its therapeutic use is of greater clinical relevance. Mice treated with type I IFN post-IAV infection showed a successful reduction in lung IAV titer but displayed increased morbidity and mortality in comparison to vehicle-treated mice (222). A possible explanation for this phenomenon is the induction of excessive inflammatory response and TRAIL-DR5-mediated epithelial cell death by type I IFN (223), which accounts for the observed lung pathology in IAV-infected animals treated with type I IFN (224). In addition, downregulation of γδ T-cells by type I IFN has been correlated with increased susceptibility to secondary *S. pneumoniae* infection (225), further arguing against the potential use of type I IFNs for the treatment of IAV infection.

In comparison to type I IFNs, the administration of type III IFNs may provide advantages in the control of IAV replication (176, 222, 224) without the risk of previously reported type I IFNs-mediated immunopathologic side-effects (222, 224, 226). However, a recent study aiming to stimulate IFNs signaling through the systematic administration of RIG-I ligand post-IAV infection demonstrated that type I, but not type III IFNs signaling is important in conferring protection during fatal IAV infection *in vivo* (227). Though, this study did not measure the production of type I and III IFNs as well as any changes in viral load with respect to *Ifnar* or *Ifnlr* knockout. In addition, while human immune cells are not primary targets in IAV infection, they could be susceptible to IAV and become efficient host cells for virus replication. They are reported to possess a subpar response to type III IFNs (222); leading to the preliminary conclusion that solely using type III IFN as treatment may not be feasible. As such, reports suggesting the use of type III IFNs over type I IFNs as a front-line therapeutic agent to counter IAV infections may require further investigation.

#### Prostaglandin E_2_

The inhibition of COX-2 by selective inhibitors, nimesulide and celecoxib, was previously demonstrated to suppress the hyper upregulation of pro-inflammatory cytokines induced by highly pathogenic avian IAV (228–230). In addition, the use of zanamivir in tandem with a specific COX-2 inhibitor was shown to increase the survival rate of mice lethally infected with avian H7N9 IAV, when compared to mice treated solely with zanamivir (229).

Activated COX-2 regulates downstream prostaglandin production. One such example is PGE_2_, a major type of prostaglandin recently demonstrated to play an important role during IAV infection. PGE_2_ was significantly upregulated in response to IAV infection, leading to the inhibition of antiviral type I IFN production in macrophages and the subsequent increase in virus replication (231). The use of chemicals AH6809 and GW627368X to antagonize PGE_2_ downstream signaling molecules EP2 and EP4 respectively, was shown to induce antiviral type I IFN production. The *in vivo* treatment of mice lethally challenged IAV with both EP2 and EP4 antagonists significantly improved the survival rate.

A recent study demonstrated the ability of a modified TCM decoction to reduce PEG_2_ production and subsequent morbidity in mice lethally challenged with IAV. Improved lung pathology was observed (232). The long history of clinical TCM use supports the clinical feasibility of PEG_2_ inhibition as an option to treat severe IAV infections.

#### Toll-Like Receptors (TLRs)

Pattern recognition receptors on host cells sense specific PAMPs present on the viral surface or generated during replication. PRRs can be broadly divided into two classes by their function or location. When defined by location, PRRs are classified into 3 groups—membrane-bound (TLRs and C-type lectin receptors), cytosolic (RIG-I-like and NOD-like receptors), and secreted (collectins and pentraxins) (233).

Significant research has been conducted on PRRs with regards to IAV infection. TLRs and RIG-I receptors have been extensively studied for their major roles in eliciting host immune responses (cytokine and IFN expression) during IAV infection (234–236). RIG-I receptors have been investigated for their functional relevance to IAV infection and targeting these receptors as a form of IAV treatment has been extensively reviewed (237–239). This section will cover recent research on TLRs and the targeting of different TLRs to treat IAV infection.

Humans have been identified to express TLR1–10, while mice have been identified to express functional TLR1–9 as well as TLR11–13 (240). Most TLRs—with the exception of TLR3—utilize MyD88 as an adaptor protein during signal transduction. TLR3 utilizes TRIF as an adaptor. TLR4 is known for its ability to utilize either MyD88 or TRIF, with the choice of adaptor dependent on its sub-cellular location (241). Different TLRs, such as TLR3, 7, and 8 (240) as well as TLR2, TLR4, and most recently TLR10 (235), have been revealed to play a role in the orchestration of host immune responses contributing to IAV pathogenesis.

With TLR10 being an exception (242–244), TLR activation largely causes the release of pro-inflammatory cytokines, with hypercytokinemia leading to ALI as a major cause of mortality in severe IAV infections. In addition to dysregulated cytokine release, excessive production of ROS has been associated with ALI development. In fact, lung injury during severe pulmonary infections, such as IAV and SARS, could be caused by oxidative stress (245). IAV infection activates NADPH oxidase that subsequently produces oxidized PAPC, an endogenous phospholipid. The oxidized PAPC serves as an agonist for TLR4, activating a TLR4-TRIF-TRAF6-NF-κB signaling cascade to eventually trigger the release of IL-6, ultimately inducing the onset of ALI. In addition to oxidized PAPC, the induction of endogenous protein S100A9 upon intracellular PRR DDX21 recognition of IAV subsequently induces the activation of TLR4, further contributing to IAV-induced mortality (246). Since TLR4 has been proven to be important in ALI induction (and hence IAV-related mortality), manipulating the stimulation and antagonism of TLR4 could potentially reduce the severity of IAV infections.

Eritoran (E5564) is a specific TLR4 antagonist initially purposed for the treatment of sepsis, but a failed a phase III clinical trial due to improved patient care in the placebo group prevented its eventual use in sepsis treatment (247). *In vivo* administration of eritoran in mice lethally infected with IAV resulted in improved clinical score, lung pathology results, and reduced viral titer. Delayed administration of eritoran, at day 6 after infection beyond the recommended therapeutic time window (within 48 h after the first display of clinical symptom) for use of oseltamivir (248), also demonstrated a significant benefit to infected mice compared to non-treated group, suggesting a prolonged therapeutic time window for IAV treatment when compared to mainstay antiviral drug treatment. A newer and structurally simpler specific TLR4 antagonist, FP7 (249), alongside a newly developed decoy peptide 2R9 that has been shown to disrupt TLR2, 4, 7, and 9 signaling *via* TIRAP, has been shown to protect mice from lethal IAV infection (250). These results support the potential use of TLR4 antagonism as a means to treat severe IAV infection.

The suppression of other TLR signaling pathways—such as blocking TLR2-mediated signaling through the use of an anti-TLR2 antibody, significantly protected against lethality when administered on day 2 and 4 post-IAV infection (251).

A study also demonstrated that H5N1-infected TLR3 knockout mice had better survival than H5N1-infected wild-type mice, which is evident through the significantly faster regaining of body weight post-infection, lower viral titer in the lung, and fewer pathological changes in the lung (252).

An increasing number of TLR antagonists are now under development (253, 254), alongside several other agents also shown to have effects on TLRs. Polysaccharides isolated from *R. isatidis*, a traditional Chinese medicinal herb used to treat IAV infection, have recently been shown to inhibit pro-inflammatory cytokines such as IL-6 and CCL-5 *in vitro* by down-regulating upstream TLR3 expression (255). MENK, an endogenous protein expressed in the adrenal medulla, was shown to both prophylactically and therapeutically increase the survival rate while reducing viral-caused lung pathology and viral titer in mice lethally challenged with IAV (256). This was determined to be caused by the downregulation of TLR7. These results suggest the potential of down-regulating TLR expression in the treatment of IAV infection.

The above-mentioned data suggest modulation of TLR signaling or expression as a promising approach in treating severe influenza disease and deserves immediate investigation. Table 2 summarizes new immunomodulatory approaches to combat IAV infections.

**Table 2:** New immunomodulatory approaches to treat influenza A virus (IAV) infection.

Potential target/approach	Possible intervention	Effect	Reference
Histones	Anti-histone antibodies	Prevents lung inflammation and damage induced by histones	Ashar et al. (161)
NETosis	Cl-amidine Tetrahydroisoquinolines (both tested on NETosis, effect on IAV yet to be determined)	Prevents lung injury mediated by NET	Kusunoki et al. (164) Martinez et al. (163)
ILC2	To be determined	Promotes airway epithelium repair	Califano et al. (171)
Nox2	Cholestanol-conjugated gp91ds-TAT Apocynin [also as reactive oxygen species (ROS) scavenger] Ebselen (also as ROS scavenger and glutathione peroxidase mimetic)	Inhibits Nox2 activity	To et al. (177) Ye et al. (154) Oostwoud et al. (184)
Tumor necrosis factor (TNF)	Etanercept	Prevents TNF-mediated lung injury and edema	Shi et al. (205)
IL-6	Administration of IL-6	Inhibits cell death of neutrophils; limits CD8^+^ T-cell-induced lung injury	Dienz et al. (206); Pyle et al. (208)
IL-27	Administration of IL-27 at recovery phase (5–10 days post-infection)	Promotes recovery and improves lung immunopathology	Liu et al. (214)
Type III IFN	Administration of type III IFN	Controls IAV replication by type III IFN signaling pathways	Davidson et al. (222); Kim et al. (224)
PGE_2_ signaling	AH6809 (EP2 antagonist) GW627368X (EP4 antagonist)	Restores type I IFNs induction which are suppressed by PGE_2_ in macrophages	Coulombe et al. (231)
TLR4 signaling	TLR4 antagonists (a. Eritoran and b. FP7)	Ameliorates TLR4-mediated lung injury	Shirey et al. (248) Perrin-Cocon et al. (249)
TIRAP antagonism	Peptide 2R9	Inhibits multiple TLR signaling	Piao et al. (250)
TLR2 signaling	Anti-TLR2 antibody	Inhibits TLR2 signaling mediated lethality	Shirey et al. (251)
TLR3 signaling	Polysaccharides isolated from *R. isatidis*	Down regulates TLR3 expression to inhibit hypercytokinemia	Li et al. (255)
TLR7 signaling	MENK	Down regulates TLR7 expression and reduces lung pathology	Tian et al. (256)

## Modulation of Metabolism

It is well documented that patients with diabetes mellitus have a greater tendency to develop severe IAV infection than healthy patients (257). Hyperglycemia increases susceptibility of the host to IAV infection *via* viral uptake, through the promotion of V-ATPase assembly (258) and immunosuppression (257). In addition, viruses rely on host metabolism to perform essential functions during replication (259–262). These processes exert a large energy demand on the host within a very short period of time (263); energy of which is supplied by and is dependent on host metabolism. IAV viruses have been reported to modify the metabolic state of the host. For example, increased c-Myc-dependent glycolysis and glutaminolysis has been demonstrated in infected cells (264). The changes in glucose and glutamine metabolism were reversed upon the addition of BEZ235, which inhibited the IAV-mediated c-Myc induction. Administration of BEZ235 2 days prior to infection and up to 4 days post-infection was shown to decrease lung viral titer and improve the survival rate in IAV-infected mice. Small molecules such as clotrimazole and α-mangostin that target lipid metabolism have also been demonstrated to suppress IAV replication *in vitro* (264).

In addition to being important for generating energy and biosynthesis, recent research demonstrates that cellular metabolism affects immune cell function. Dysregulated immune responses observed in many diseases are associated with specific metabolic configurations. Viruses, influenza inclusive (265), were found to induce drastic alterations in metabolic levels and programs (263). Macrophages in infected hosts were observed to have marked differences in the Krebs cycle, a key metabolic pathway. This is of significance due to the role of macrophages, which are immune cells critical in the pathogenesis of many inflammatory diseases (263, 265, 266).

In activated macrophages, succinate, a Krebs cycle intermediate, was found to possess inflammatory signal. Accumulation of succinate generates ROS, leading to subsequent activation of hypoxia-inducible factor 1α and the induction of cytokines such as IL-1β (267). A recent study identified the ability of itaconate, another Krebs cycle-derived metabolite, to block the production of inflammatory factors. This prevented inflammation, protecting mice from lethal levels of inflammation that can occur during infection (268). This data suggest the critical roles of Krebs cycle intermediates in regulating cytokine profiles and inflammation. Metabolites generated by innate immune cells in distinct configurations could have different roles beyond that of bioenergetics, with functions in signaling regulation, transcription, and orchestrating innate immune responses.

Despite the lack of research conducted thus far on the application of immunometabolic approaches to influenza treatment, the prospect of manipulating immune responses by modulating immune cell metabolic state is promising. Further research should focus on the identification of metabolites for modulation of immune cell function with substantial improvement of therapeutic strategies to treat IAV disease.

Latest advancements in high-throughput technologies, e.g., metabolomics is a useful approach to systematically investigate the changes of metabolic mechanisms during IAV infections. Identification of important metabolites involved during IAV infection should be a new approach by modulating the host metabolism for interventions.

## Concluding Remarks

Multiple host-based intervention strategies against influenza have been developed or are under development. While approaches targeting host machinery required for virus replication seem to be promising thus far, additional research is needed to determine the effect of modulating host immune response on influenza treatment. This is increasingly important, since targeted host factors may play distinct roles in response to infection by different influenza viral strains (252), making the management of influenza through solely targeting a single specific host factor is difficult.

Host-based interventions offer obvious advantages over conventional antivirals, such as a higher barrier to drug resistance (73, 83, 107) due to greater genetic stability of host factors than the mutation-prone nature of viral components. In addition, administration feasibility is a key factor to consider the usage of drugs. The mainstays of antivirals for IAV infections, the NA inhibitors, and M2 blockers, are recommended to be administered within 48 h of symptom onset for optimal antiviral activity. This short treatment window may not be fully fulfilled in a clinical setting. Novel host-based interventions were reported to have therapeutic time windows longer than this conventional timeframe (96, 109, 214, 251), even up to 6 days post-infection (248), providing a clear clinical advantage over NA inhibitors and M2 blockers. In addition, hypercytokinemia and ARDS could contribute to disease severity and mortality in instances of severe influenza infection, with virus-targeting antivirals providing little to no alleviation of such complications.

Since host immune response is indispensable in host defense against invading pathogens, the use of immune-modulators to suppress detrimental effects while retaining beneficial protection of the host remains challenging. The timing and dosage of medication administration would be critical in determining the drug effectiveness in influenza treatment.

Targeting virus-induced metabolic changes to restore host normal metabolism may be a new direction to combat influenza disease. Further research in the immunometabolism field, alongside studies on modulating immune response to infectious disease by altering host metabolic processes; would create a new direction for future research and is expected to yield significant discoveries that may provide new therapeutic options in the treatment of IAV infections.

## Author Contributions

SMYL conceptualized the work. IL and ASMS drafted some review sections and TFY and SMYL wrote the manuscript.

## Conflict of Interest Statement

The authors declare that the research was conducted in the absence of any commercial or financial relationships that could be construed as a potential conflict of interest.
